# Breast cancer survival prognosis using the graph convolutional network with Choquet fuzzy integral

**DOI:** 10.1038/s41598-023-40341-z

**Published:** 2023-09-07

**Authors:** Susmita Palmal, Nikhilanand Arya, Sriparna Saha, Somanath Tripathy

**Affiliations:** Department of Computer Science and Engineering, Indian Institute of Technology, Patna, Bihar 801106 India

**Keywords:** Breast cancer, Machine learning

## Abstract

Breast cancer is the most prevalent kind of cancer among women and there is a need for a reliable algorithm to predict its prognosis. Previous studies focused on using gene expression data to build predictive models. However, recent advancements have made multi-omics cancer data sets (gene expression, copy number alteration, etc.) accessible. This has acted as the motivation for the creation of a novel model that utilizes a graph convolutional network (GCN) and Choquet fuzzy ensemble, incorporating multi-omics and clinical data retrieved from the publicly available METABRIC Database. In this study, graphs have been used to extract structural information, and a Choquet Fuzzy Ensemble with Logistic Regression, Random Forest, and Support Vector Machine as base classifiers has been employed to classify breast cancer patients as short-term or long-term survivors. The model has been run using all possible combinations of gene expression, copy number alteration, and clinical modality, and the results have been reported. Furthermore, a comparison has been made between the obtained results and different baseline models and state-of-the-art to demonstrate the efficacy of the proposed model in terms of different metrics. The results of this model based on Accuracy, Matthews correlation coefficient, Precision, Sensitivity, Specificity, Balanced Accuracy, and F1-Measure are 0.820, 0.528, 0.630, 0.666, 0.871, 0.769, and 0.647, respectively.

## Introduction

Breast cancer is a very heterogeneous disease that affects women of different ages. The breast consists of different tissues such as fatty tissue and dense tissue, comprising lobes, lobules, and milk glands. Breast cancer occurs when breast cells multiply uncontrollably, leading to tumor formation. If breast cancer spreads to other organs, it becomes metastatic. There are two biological types: invasive breast cancer, which spreads to adjacent tissues or distant organs, and non-invasive breast cancer, which remains confined to the lobuler portion of breasts. According to the report given by WHO, there were 6,85,000 deaths worldwide and 2.3 million new cases of breast cancer in women in 2020. Therefore, early prognosis prediction is necessary for more specific therapy and treatment. In this context, calculating a patient’s likelihood of survival is a significant problem with cancer prognosis prediction^1^. It can be described as a censored survival analysis issue, which is used to determine whether and when an event (like a patient’s death) will occur within a given time frame^2^. The five-year survival rate is a frequently used statistic for the prognosis of breast cancer^3^. Prognostication of a patient’s life expectancy is crucial because when a sufficiently accurate prognosis estimate is supplied, it helps in clinical decision-making^4,5^. Moreover, breast cancer is a complex disease with diverse clinical outcomes, making prediction and treatment challenging. Such as the high dimensionality of multimodal data poses difficulties for medical professionals in manual interpretation. Hence, the development of computational algorithms becomes crucial in accurately predicting the prognosis of breast cancer. These algorithms can assist physicians in determining the most appropriate treatment plan for patients, emphasizing the significance of such approaches in clinical decision-making.

## Related works

In past studies, the problem of forecasting a cancer patient’s prognosis was addressed by many researchers. However, due to the complexity or implausibility of combining many sources, many prognostic prediction studies typically rely on a single type of data. Such as Xu et al.^6^ developed a prognosis prediction system using support vector machines by including a recursive feature reduction strategy based on gene expression data. Furthermore, Nguyen et al.^7^ created a prediction model with random forest (RF) to select features that outperform the pre-existing Wisconsin Breast Cancer Prognostic model. Yet, multi-modal modeling techniques have demonstrated that incorporating many forms of data can improve forecast accuracy. Regarding multi-modal data, Sun et al.^4^ have suggested a methodology that employs a hybrid signature made up of Clinical and Gene expression data. Further, a probabilistic model which includes Bayesian Network was proposed by Gevaert et al.^5^. They considered genetic and clinical data for their study. Moreover, the recent advancements in deep learning methodologies showed that the models which use data with multiple modalities often perform better than uni-modal models. Sun et al.^8^ established this fact by constructing a deep neural network-based model by incorporating multi-omics and clinical data. Furthermore, Sun et al.^9^ presented the GPMKL model for determining the breast cancer survival prognosis by integrating pathological images with genomics data. Hsu et al.^10^ also combined gene expression data with clinical data for the breast cancer survival prediction study. Further, Arya et al.^11–15^ also introduced some new models by incorporating deep learning-based architecture for this study. However, predicting cancer survival using clinical and multi-omics data remains challenging due to limited feature size and complex structured data. Therefore, thoroughly exploring clinical and multi-omics data using various machine-learning techniques is crucial for accurate prognosis estimation in cancer research. Here the details of some of the breast cancer survival prognosis prediction studies in recent past years have been depicted in Table 1. The full name of the modalities CLN, GE, HistoIm, CNA, DNAMe, and mRNASeq are clinical data, gene expression data, histopathology image, copy number alteration data, DNA-methylation data, and mRNA sequence respectively.

**Table 1:** Details of some of the breast cancer survival prediction studies in recent past years.

Author	Year	Modality	Prediction model
Gevaert et al.^5^	2006	CLN, GE	Bayesian network
Sun et al^4^	2007	CLN, GE	Correlation based classifier
Xu et al.^6^	2012	GE	SVM with a recursive feature reduction
Nguyen et al^7^	2013	Image	Random forest (RF)
Sun et al.^9^	2018	GE, HistoIm	GPMKL (multiple kernel learning )
Sun et al.^8^	2018	CLN, GE, CNA	Multimodal deep neural network
Arya et al.^12^	2020	CLN, GE, CNA	Deep-learning based STACKED ensemble
Hsu et al.^10^	2021	CLN, GE	Ensemble learning with bimodal DNN
Arya et al.^11^	2021	CLN, GE, HistoIm.	Gated attentive deep learning with RF
Arya et al.^13^	2021	CLN, GE, CNA	Generative incomplete multi-view prediction (GIMPP)
Arya et al.^14^	2022	CLN, GE, CNA, HistoIm.	SVM based on utility kernel
Arya et al.^15^	2023	CLN, GE, CNA, DNAMe,	PCA and VAE with
		mRNASeq, HistoIm	RF and SVM
Du et al.^16^	2023	CLN, GE, CNA	Multi-scale bilinear convolutional neural network

## Motivation for the proposed model

Even if the aforementioned deep learning-based methods for predicting cancer survival have produced promising results, the subsequent area still has a lot of room for growth. The question of exploiting the structural information between samples’ underlying linkages has not been addressed in prior studies. To address this issue, considering muti-omics and clinical data, a novel study using Graph Convolutional Network with a Choquet Fuzzy Ensemble (ChoqFuzGCN) has been proposed for the survival prediction of cancer patients. It is also observed that there exist certain drawbacks in some of the latest proposed models. For instance, the authors of a recent work^11,12^ utilized a Convolutional Neural Network (CNN)^17^ for feature extraction as it can generate a comprehensive stacked feature set by producing a large number of hidden features from input data. However, CNNs are limited in that, they only consider data points that are neighboring based on the kernel size and do not account for other data points that may have high correlations but are not directly connected. This is a significant limitation of their approach. Whereas, graph convolution takes into account the irregular structure of data and selects neighboring nodes for information sharing and convolution based on their correlations. Notably, the number of neighboring nodes can vary depending on a data point’s connectivity with other nodes, making information-sharing dynamic and independent of fixed kernel size. Moreover, typically, a variety of ensemble learning techniques lead to positive predictive outcomes^18^. Such as, after examining state-of-the-art model MDNNMD^8^, it has been noticed that a classifier level ensemble has been utilized to compute the result. This is a simple method where each classifier in the ensemble makes a prediction, and the end result is based on the weighted vote of the classifiers. But in the proposed model, the utilization of the Choquet fuzzy integral has been considered to use the classifier’s probabilistic results for producing the end predicted values. The Choquet fuzzy integral is a more sophisticated approach that can produce more accurate results by taking into account the degree of agreement among the classifiers and handling non-linear relationships among them. Recent study ^19^ has provided compelling evidence that fuzzy-based ensemble techniques outperform simple ensemble techniques.

In this work, graphs have been created using different modalities of data. The graphs investigate the natural correlations between samples or patients. Also, embedding representation for all samples was computed by applying the graph convolution technique^20^ to each graph. After obtaining the feature embedding of each sample, a Choquet Fuzzy Integral-based ensemble^21^ of three base classifiers, namely Random Forest (RF)^22^, Support Vector Machine (SVM) with Radial Basis Function (RBF) Kernel^3^, and Logistic Regression (LR)^23^, has been applied. This fuzzy ensemble uses the probabilistic outcomes of different classifiers to obtain the prediction value. Here a threshold value (5 years) has been chosen to make the distinction between the two prediction classes. Patients who survived more than five years are considered long-term survivors whereas patients who survived lesser than five years are considered short-term survivors.

In brief, the contributions made by the current study are as follows:

Contribution I: From single to multi-omics approach: The aim of addressing whether transitioning from a single-modality approach to a multi-omics approach works well has been pursued. An extensive study has been performed using Clinical (*CLN*), Copy Number Alteration (*CNA*), and Gene expression (*GE*) data. To compare the performance improvement, experimental results using multi-omics and clinical data have been reported. Also, the results based on individual modality have been reported.

Contribution II: Novel method: In this work, a Graph Convolutional Network (GCN)^20^ based prediction model has been proposed to extract the important features of multi-omics data. Furthermore, an ensemble of base classifiers based on Choquet Fuzzy Integrals has been suggested, which utilizes the probabilistic results of each classifier to produce the final prediction. The Choquet Fuzzy Integral-based ensemble takes advantage of the level of uncertainty that exists in the decision scores and improves the prediction performance.

## Dataset

The pre-processed version of the publicly available METABRIC dataset (https://www.cbioportal.org/study/summary?id=brca_metabric) has been taken for this study. The pre-processed version of the dataset is available on Github: (https://github.com/USTC-HIlab/MDNNMD). This dataset consists of three different modalities, namely, Gene Expression data (*GE*), Copy Number Alteration (*CNA*) profile, and Clinical data (*CLN*), and each has around 24000, 26000, and 27 features, respectively. The dataset has 1980 samples. Among these, there are 491 short-time survivors and 1489 long-time survivors. Here, survivors with fewer than five years are referred to as short-time survivors, and those with more than five years are referred to as long-time survivors. Features from different modalities play a crucial role in capturing distinct biological and clinical information. GE features highlight coordinated gene expression changes based on different classes like cancerous or non-cancerous. CNA profiles provide insights into genomic alterations, such as amplifications or deletions, aiding in the identification of important genes. On the other hand, CLN data, encompassing patient demographics and clinical variables, offer contextual information that complements molecular data and enables personalized prognosis and treatment decisions. Integrating these diverse features enhances predictive models and improves disease understanding.

### Data pre-processing

Each patient in the study is associated with 27 clinical features, including variables such as age at diagnosis, tumor size, and lymph nodes positive. From these clinical features, a final set of 25 features is selected for analysis. To address missing values in the GE profile and CNA profile data, a weighted nearest neighbor algorithm was employed for estimation. The GE features were normalized and discretized into three categories: under-expression ($-1$), over-expression (1), and baseline (0). For the clinical data, all features are normalized using min-max normalization to a range of [0, 1]. The CNA features are utilized in their original form, consisting of five discrete values ($-2, -1$, 0, 1, 2). These preprocessing steps ensure consistency and appropriate scaling for the different types of data used in the analysis. Furthermore, the “curse of dimensionality” poses a challenge in human cancer prognosis prediction using high throughput sequencing datasets. The dataset utilized in this study consists of approximately 24,000 genes for gene expression profiles and 26,000 genes for CNA profiles. To address the high dimensionality and small sample size, mRMR^24^ feature selection is applied to reduce dimensionality while preserving information. Evaluation is performed using the area under the curve (AUC) value, and a search is conducted to select the best N features (100 to 500, step size 100). Ultimately, 400 genes from gene expression profiles and 200 genes from CNA profiles are chosen.

## Methodology

**Figure 1:**
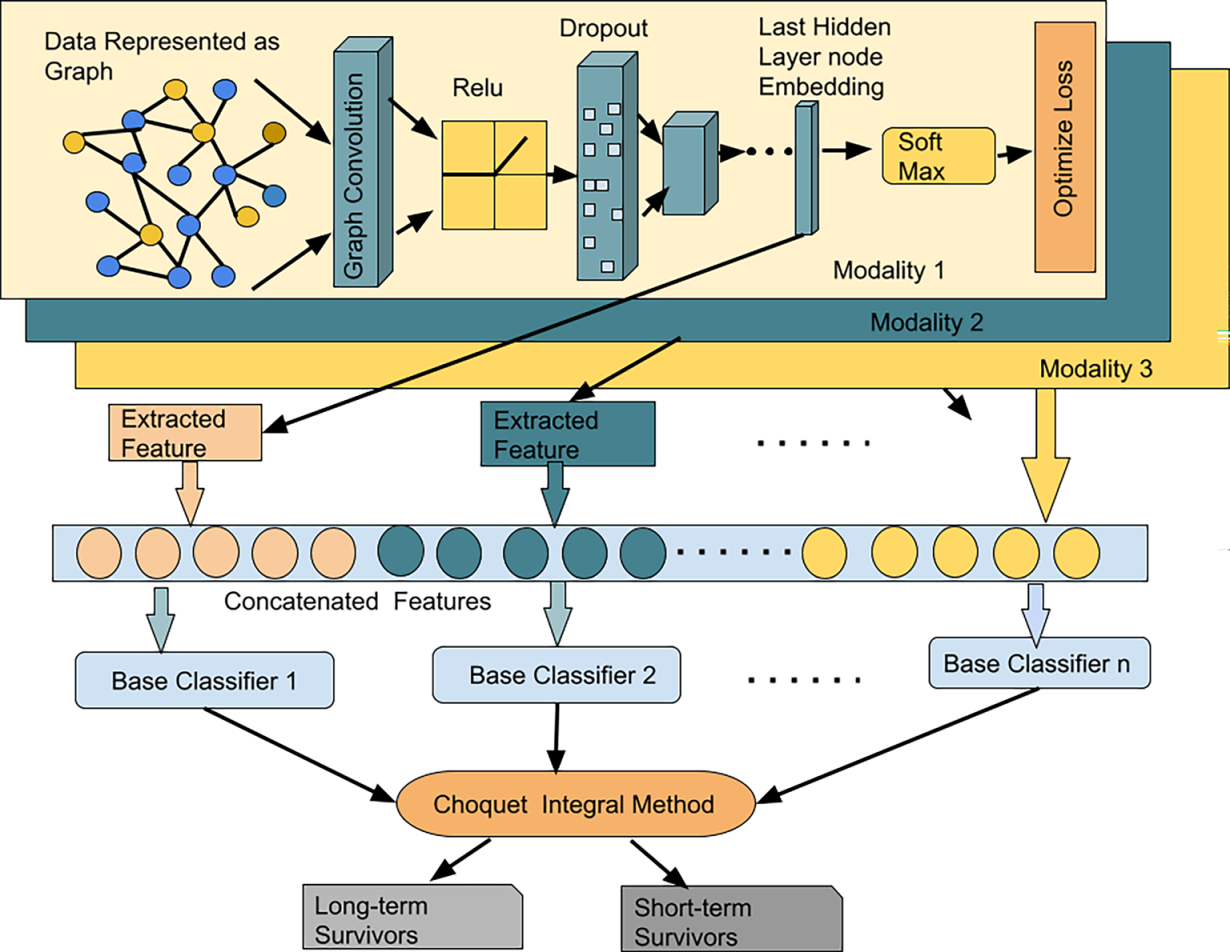
Proposed architecture (ChoqFuzGCN).

In this proposed work (Fig. 1﻿), a Graph Convolutional Network (GCN) based feature extraction method has been created with a Choquet Fuzzy Ensemble of base classifiers (RF^22^, SVM with Radial Basis Function (RBF) Kernel^3^, LR^23^) for breast cancer patient categorization into long-term and short-term survivors. Different graphs have been constructed for incorporating structural relationships among data instances. For the construction of the graph, data instances are considered as nodes and edges have been formed by computing correlation among the instances. A graph has been formed for gene expression data (GE). Also, another graph was formed for copy number alteration data (CNA). Here, based on experimental findings, a certain correlation threshold (0.3 for *GE* and 0.6 for *CNA*) has been chosen to create an edge between any two nodes. In this case, Pearson correlation^25^ has been used. Furthermore, convolution^20^ has been applied to each of the graphs. This convolution technique helps to learn the irregular structure of the data which may be present among connecting nodes. Then, the end node embedding has been recovered, and the feature vector has been retrieved following the completion of the training of the model using GCN. The feature vectors from the various modalities have been combined or stacked to generate the final feature vector. Here, GCN struggles to provide proper node embedding for Clinical data because it has a substantially smaller number of characteristics. Therefore, to build the stacked feature set, clinical data has been directly concatenated with the derived features from the other two modalities. The final classification has been carried out via an ensemble of base classifiers using Choquet Fuzzy Integrals^21^. The selection of these base classifiers is primarily motivated by their widespread usage in state-of-the-art^15,26^ methods within the field. Random Forest (RF)^22^ works by constructing multiple decision trees using bootstrapped samples from the dataset and aggregating their predictions through voting or averaging, resulting in a robust and accurate classification or regression model. Logistic Regression^23^ works by fitting a logistic function to the input data, estimating the probability of binary outcomes, and making predictions based on a specified decision boundary. On the other hand, SVM with RBF^3^ kernel is a valuable approach when dealing with datasets that exhibit nonlinearity, as it employs a transformation to a higher-dimensional space, enabling the identification of an optimal hyperplane that maximizes the separation between distinct classes.

This study presents an original contribution by introducing a prediction model that utilizes a Graph Convolutional Network (GCN) to extract important features from multi-omics data. Moreover, a novel ensemble method based on Choquet Fuzzy Integrals (CGI) is proposed, which combines the probabilistic outputs of individual classifiers to produce the final prediction. By leveraging the inherent uncertainty in the decision scores, the Choquet Fuzzy Integral-based ensemble enhances the prediction performance. The CGI is considered a dynamic technique as it enables the dynamic integration and aggregation of contributions of base classifiers in the final ensemble. It takes into account the varying levels of uncertainty, importance, or relevance associated with each base classifier. Therefore, this model not only captures essential features from multi-omics data using GCN but also improves predictions through the integration of Choquet Fuzzy Integrals, effectively leveraging probabilistic outputs and enhancing overall accuracy.

### Graph convolutional network (GCN)

In a convolutional neural network (CNN), kernels are used for learning the features of the neighboring cells by moving over the entire dataset. As the shape of the kernels remains fixed, CNN is able to learn the neighboring features by considering the dataset as a regular structure. However, to explore the nonregular pattern which may exist in a dataset, CNN is not a helpful technique. In such case, graph convolutional networks^20,27^ are able to handle irregular data patterns. It is a semi-supervised technique. These networks consider non-Euclidean structured data and leverage the examination of neighboring nodes to extract features. Consider a graph G with the sets of nodes and edges V and E, respectively. In this instance, the adjacency matrix, represented as *A*, stands for the edge connections between nodes. The forward pass equation (Eq. 1) for the kth hidden layer of GCN is:

1 $$\begin{aligned} L^{[k+1]}= \sigma (A^*L^{[k]}W^{[k]}) \end{aligned}$$

Here $A^*$ stands for the normalized version^20^ of *A*, $L^{[k]}$ represents the *k*th hidden layer, $W^{[k]}$ denotes the weight matrix, $\sigma$ stands for the activation function (ReLU). Here, the node feature matrix is represented by *X*. By computing the dot product of *A* and *X*, the model learns all the feature values for each node from neighbors, referred to as *AX*. Further, it is necessary to normalize the features to prevent vanishing/exploding gradients in the model convergence. To achieve this, data is normalized by calculating the Degree Matrix (D) and performing the dot product operation of the inverse of *D* with *AX* (Eq. 2).

2 $$\begin{aligned} Norm\_features=D^{-1}AX \end{aligned}$$

Here, the number of linked edges on a specific node is called its “degree.” Further, the obtained symmetric normalization equation^20^ (Eq. 3) from normalization equation (Eq. 2) is as follows:

3 $$\begin{aligned} Norm\_features=D^{-1/2}A D^{-1/2}X \end{aligned}$$

So, for the first hidden layer of GCN, the forward pass equation (Eq. 4) is:

4 $$\begin{aligned} L^{[1]}= \sigma (D^{-1/2}A D^{-1/2}L^{[0]}W^{[0]}) \end{aligned}$$

Here, D^-1/2^AD^-1/2^=A*, L^[0]^=*X,* and W^[0]^ is the weight matrix. Likewise, the forward pass equation of kth hidden layer is given in equation 1. The loss function is calculated by the cross-entropy error over all labeled examples. This is explained in the subsection ‘Objective function’.

### Choquet fuzzy integral

In this study, the ensemble of three machine learning base classifiers (logistic regression^23^, support vector machine^3^, and random forest^22^) has been proposed, utilizing the Choquet fuzzy integral method^21^. The pre-eminence of individual base classifiers towards the final prediction goal is determined with the help of this approach. The input of the fuzzy fusion is constituted by the confidence ratings obtained from various classifiers. In this fusion approach, the decision scores’ uncertainty, which refers to the level of confidence or uncertainty associated with the predictions made by a classifier, is considered as an additional piece of information that is available for the fusion process.

Let $C_1$, $C_2$, and $C_3$ be the three base classifiers representing Logistic Regression, Support Vector Machine, and Random Forest, respectively. Here, $C_j$ and $score_j$ denote the j$_{\textrm{th}}$ classifier and its validation accuracy, with $1 \le j \le 3$. As fuzzy measure values determine the strength of individual classifiers as well as combinations of classifiers, we must determine the fuzzy measure values for each classifier before we can utilize the Choquet fuzzy integral. $fuzz(\{C_j\})\in$ [0,1] represents the fuzzy measure of j$_{\textrm{th}}$ classifier and calculated as ${(score_j)}/{(\sum \limits _{j=1} ^3 score_j})$. The $fuzz(\{C_j\})$ has two boundary cases as follows:

1. If all classifiers are present in the combination (represented by set *S*), it will have maximum pre-eminence, i.e., $fuzz(S)=1$.
2. If no classifier is present in the combination, then it will have no pre-eminence, i.e., $fuzz(\phi )=0$

Further, to get the fuzzy measures of various combinations of classifiers, we need to calculate the value of $\lambda > -1$ using the theory of fuzzy integral^21^ as follows:

5 $$\begin{aligned} 1 + \lambda = \prod \limits _{i=1} ^3 (fuzz(\{C_i\})\lambda +1) \end{aligned}$$

After solving Eq. (5), we will have the value of $\lambda$, which can be further utilized in getting fuzzy measures of any possible combinations of classifiers. It can be calculated using an equation under the assumption that the two subsets (*X*, *Y*) of classifiers are mutually exclusive (i.e., $X \cap Y = \phi$) as follows:

6 $$\begin{aligned} fuzz(\{C_l, C_o\}) = fuzz(\{C_l\}) + fuzz(\{C_o\})+ \lambda fuzz(\{C_l\}) fuzz(\{C_o\}) \end{aligned}$$

where, $$1 \le l,o \le 3$$.

Once the fuzzy measures and performance scores of all the base classifiers are computed, Choquet fuzzy integral can be used to ensemble these scores to get the final prediction score. Let, $C=\{C_{1}, C_{2},\ldots , C_{m}\}$ be the set of *m* classifiers with $SCORE =\{score_{1},score_{2}, \ldots , score_{m}\}$ as their respective performance scores such that $score_{1}\le score_{2}\le \cdots \le score_{m}$. The fuzzy measures over these classifiers are {$fuzz(A_1), fuzz(A_2), \ldots , fuzz(A_m)$}, where $fuzz(A_i) = fuzz(\{C_{i},C_{i+1},\ldots , C_{m}\})$. Then the Choquet fuzzy integral is defined as follows:

7 $$\begin{aligned} Choquet_{fuzz}(SCORE) = \sum _{i=1}^{m} (score_{i}-score_{i-1}) fuzz(A_{i}) \end{aligned}$$

where $$score_0 = 0$$.

### Steps of proposed technique

The following steps show the suggested method’s working mechanism.

1. In this research, separate graphs are constructed for the gene expression (*GE*) and Copy Number Alteration (*CNA*) modalities, where samples are represented as nodes. The presence of an edge between two nodes is determined based on the Pearson correlation exceeding a specific threshold value (0.3 for Gene expression and 0.6 for *CNA* ) derived from experimental observations.
2. Within the dataset, the 1980 samples are randomly divided into 10 subgroups. Out of these subgroups, 9 are combined and utilized as the training set, while the remaining subset is sequentially employed as the testing set.
3. To establish a validation set, 20% of the data is extracted from the training set. Initially, individual GCN models are trained for the *CNA* and *GE* data separately to determine the optimal parameters.
4. The final node embeddings of the *CNA* and *GE* modalities are extracted independently using GCN, while GCN is not employed with clinical data (*CLN*) due to its limited number of attributes, which is insufficient for constructing a graph to convey structural information.
5. The *CLN* features are combined with the extracted features from the final layer of the trained GCN models for the *GE* and* CNA* modalities. These features are then concatenated together for further analysis.
6. Further, up-sampling is applied using Synthetic Minority Oversampling Technique (SMOTE)^28^ on the concatenated data.
7. Following that, an ensemble of base classifiers employing Choquet Fuzzy Integrals performs the final classification^21^. As base classifiers, we have used Random Forest (RBF Kernel)^22^, Support Vector Machine^3^, and Logistic Regression^23^.

### Objective function

The proposed model is implemented using a supervised setting. The over-fitting of the model is addressed in this instance using the L2 regularization procedure. The loss function applied in this scenario is the cross-entropy loss shown in Eq. (8). In this instance, the predicted value is denoted by $\hat{y}_{p_i}$ and the actual class label by $y_{p_i}$. The batch size is *N*. $W^k$ is an abbreviation for *k*th weight matrices where $W^k=({w_{ij}}^k)_{m_k \times n_k}$. Here, there are *K* total weight matrices in existence.

8 $$\begin{aligned} L(y_p,\hat{y}_p)=-\frac{1}{N}\sum _{i=0}^{N}[y_{p_i} log (\hat{y}_{p_i})-(1-y_{p_i})log(1-\hat{y}_{p_i})]+ \frac{1}{2}{\lambda }\sum _{k=1}^{K}\sum _{j=1}^{n_k} \sum _{i=1}^{m_k}{w_{ij}} ^{k^2} \end{aligned}$$

### Computational complexity

Computational complexity for graph construction: This model incorporates graph construction using Pearson correlation computation between every pair of samples. Let’s consider training dataset size N of dimension F. So, Pearson Correlation computational complexity between any two instances with dimension D is *O*(*D*). For N data instances, the number of computations: $\{(N-1)+(N-2)+(N-3)+ \cdots +2+1\}\times O(D)=O(N^2D)$

Computational complexity of graph convolution : Consider a graph $G=(V, A, E)$, where V, A, and E are the set of vertices or instances, adjacency matrix, and set of edges respectively. Here, $N=|V|$. If $F_l$ is considered as a graph embedding representation of *l*th layer, the number of computations for matrix multiplication in a dense layer will be of size $N \times F_l$ and $F_l\times F_{l+1}$. Further, after considering $F_l=F_{l+1}=F$, the dense layer’s matrix multiplication computational complexity will be $O(NF^2)$. On the other hand, information aggregation between neighborhood nodes is done using matrix multiplication of size $N\times N$ and $N\times F$ yielding $O(N^2F)$. So overall time complexity for L number of layers is $O(LNF^2+LN^2F)$^29^. So, for I number of training iterations, it will be $O(ILNF^2+ILN^2F)$.

Computational complexity of ensemble method : Let’s, consider the number of classes is C, and the number of classifiers is M, then the complexity of the Choquet fuzzy integral is $O(C \times (M \times log(M))$^30^.

Computational complexity of the model training: $O(N^2D)+O(ILNF^2+ILN^2F)+O(C \times (M \times log(M)))$.

The overall complexity depends on the size of the training dataset (N), dimensionality (D), number of layers (L), number of classes (C), number of classifiers (M), and number of training iterations (I).

## Experimental results

We have discussed various experimental findings in this section.

### Evaluation measures

Accuracy (Acc)^31^, Precision (Pre)^31^, Matthews correlation coefficient (Mcc)^31^, Sensitivity (Sn)^31^, Specificity (Sp)^31^, F1Measure^32^, and Balanced Accuracy (Bal_Acc)^33^ values are taken into account as assessment metrics. The Matthews correlation coefficient is a more reliable metric. Only if the prediction yields accurate outcomes in terms of all true positives, false negatives, true negatives, and false positives values, then Mcc produces a high score. As one class appears significantly more frequently than the other, Balanced Accuracy has been provided because this statistic is useful when there is an imbalance between the two classes. It is the Sensitivity and Specificity’s arithmetic mean. F1-Measure is also provided a better assessment of model performance if the dataset is imbalanced.

### Experimental setup

Python (version 3.9) is utilized to implement the suggested strategy. To extract features, GCN is applied, and to predict class labels, Choquet Fuzzy Integral-based ensemble technique is used. Using the Tensorflow 2-based Steller API, the GCN model is created and trained in this experiment. The correlation thresholds of 0.6 and 0.3 were selected, respectively, to build the graphs for the data on GE and *CNA*. Ten-fold cross-validation is used to train the suggested model. These threshold values are determined empirically using the validation dataset as a base. Here, we have constructed GCN with three hidden layers containing 200, 150, and 100 units, respectively. We have used the ReLU activation function in hidden layers and the Softmax activation function in the output layer. The number of training epochs is 200, and the learning rate is 0.001. Adam optimizer has been used here. The loss function consists of binary cross-entropy with L2 regularization. Here, grid search has been used to find the optimal parameters of the base classifiers. In the case of the random forest, the parameter ‘n estimators’ (number of trees in the forest) has been set within the range of 50 to 500, while the ‘maximum depth’ and ‘minimum samples split’ parameters have been kept at their default values. It has been found that 70 is the best value for ‘n estimators’. In the case of SVM with RBF kernel, and logistic regression, default parameters have been considered for the experiment.

### Results using 10 fold cross-validation setting

**Figure 2:**
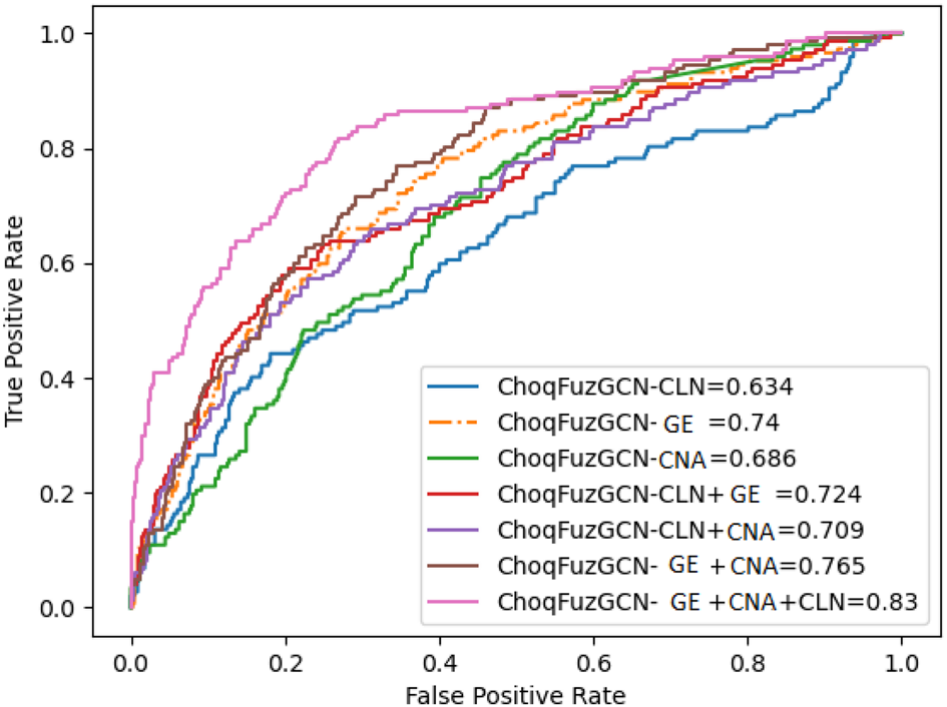
ROC curve of uni-modal and their possible combinations using ChoqFuzGCN with METABRIC data.

The outcomes of ten-fold cross-validation for the proposed model’s (ChoqFuzGCN) prediction are shown in Table 2. The testing results cover all conceivable combinations of different modalities. The last row of the table demonstrates that, in terms of the various metrics, the three combined modalities (*CLN+GE+CNA*) consistently outperformed other combinations of modalities. We also plotted the ROC curve (Receiver Operating Characteristics curve)^31^(see Fig. 2) and observed that ChoqFuzGCN with *CLN+GE+CNA* is giving better Results. The obtained AUC value with three modalities is 0.830 which is better than other combinations of modalities.

**Table 2:** Proposed (Choquet fuzzy) ensemble of base classifiers for uni, bi, multi-omics, and clinical data combinations using METABRIC dataset.

Metrics	Acc	Mcc	Pre	Sn	Sp	Bal_Acc	F1-Measure
ChoqFuzGCN-CLN	0.727	0.199	0.430	0.316	0.863	0.589	0.361
ChoqFuzGCN-GE	0.727	0.304	0.455	0.524	0.795	0.659	0.484
ChoqFuzGCN-CNA	0.726	0.180	0.424	0.283	0.872	0.577	0.337
ChoqFuzGCN-CLN+GE	0.764	0.368	0.525	0.525	0.842	0.684	0.524
ChoqFuzGCN-CLN+CNA	0.745	0.268	0.486	0.379	0.866	0.622	0.423
ChoqFuzGCN-GE+CNA	0.744	0.322	0.484	0.501	0.825	0.663	0.490
ChoqFuzGCN-CLN+GE+CNA	**0.820**	**0.528**	**0.630**	**0.666**	**0.871**	**0.769**	**0.647**

Significant values are in bold.

### Comparison with other prediction methods

The proposed model has been compared with Logistic regression (LR)^23^, Random Forest (RF)^22^, Support Vector Machine (SVM)^3^, and Majority Voting Ensemble^34^ on the extracted features from GCN layer. In the case of the Majority Voting Ensemble, we have used LR, SVM, and RF as base classifiers. Whereas, Radial Basis Function (RBF) Kernel of SVM has been used here. Table 3 presents the results in detail. It can be seen that the proposed work produced better results than other mentioned methods in terms of Accuracy, Matthews correlation coefficient, Precision, Specificity, Balanced Accuracy, and F1-Measure.

**Table 3:** Comparison of base classifiers; LR, SVM (with RBF kernel), RF, and their ensembles from the extracted feature from GCN last layer using METABRIC dataset.

Metrics	Acc	Mcc	Pre	Sn	Sp	Bal_Acc	F1-Measure
GCN-RF	0.797	0.436	0.609	0.530	**0.885**	0.707	0.563
GCN-RBF	0.778	0.490	0.539	**0.751**	0.774	0.761	0.627
GCN-LR	0.783	0.492	0.547	0.741	0.777	0.759	0.628
GCN-Majority Voting	0.786	0.490	0.552	0.725	0.806	0.765	0.626
ChoqFuzGCN	**0.820**	**0.528**	**0.630**	0.666	0.871	**0.769**	**0.647**

Significant values are in bold.

### Comparison with state-of-the-art methods

In Table 4, the performance of the proposed model has been compared with that of the existing state-of-the-art models, namely, *STACKED RF*^12^, *SiGaAtCNN+ Input STACKED RF*^11^ and * MDNNMD*^8^. *STACKED RF* has used Convolution neural network-based architecture whereas * SiGaAtCNN+ Input STACKED RF* has used sigmoid gated attention convolutional neural network. Here, all the results are reported based on 10-fold cross-validation architecture. As per the results reported by the * MDNNMD*’s work, which is deep neural network-based architecture, they have considered two separate stringency levels: Sp $=$ 99% and Sp $=$ 95%. Targeting to achieve better predictive performance, they have manually selected the classification threshold value to maintain Specificity 99% and 95%, respectively. But, in general, 0.5 is the standard binary classification threshold (i.e., if the prediction value exceeds 0.5, then the predicted class is 1; otherwise, 0). However, no additional classification threshold has been manually specified in our proposed technique, as the standard classification threshold (0.5) has been found to perform well. It can be observed in Table 4 that the performance of the proposed model has outperformed the performance of *MDNNMD* in terms of Sensitivity (0.666), Balanced Accuracy (0.769), and F1-Measure (0.647). When the stringency level is Sp $=$ 99%, ChoqFuzGCN proves its effectiveness over *MDNNMD* with 46.6%, 17.4%, and 32.1% improvements in Sensitivity, Balanced Accuracy, and F1-Measure respectively. Similarly, when the stringency level is Sp $=$ 95%, ChoqFuzGCN proves its effectiveness over *MDNNMD* with 21.6%, 6.9%, and 8.5% improvements in Sensitivity, Balanced Accuracy, and F1-Measure respectively. The comparison of the proposed model with *SiGaAtCNN+ Input STACKED RF*^11^ reveals that ChoqFuzGCN model has shown improvements of 3.2%, 14.3%, 6.5%, and 9% in Precision, Sensitivity, Balanced Accuracy, and F1-Measure values, respectively. Similarly, when compared to the STACKED RF model, ChoqFuzGCN model has exhibited performance improvements of 12.2%, 3%, and 2.5% in terms of Sensitivity, Balanced Accuracy, and F1-Measure, respectively. Furthermore, it is evident from all comparisons that F1-Measure and Balanced Accuracy are consistently superior to the existing models. Here, a high F1-Measure demonstrates that positive samples’ accurate prediction (short-time survival) is superior to the other techniques. Furthermore, the high Balanced Accuracy demonstrates that the suggested model is a superior method for addressing imbalanced data. Here, both the true positive and true negative predictions are acceptable.

**Table 4:** Performance metrics comparison with state-of-art methods with ChoqFuzGCN using METABRIC dataset.

Metrics	Pre	Sn	Sp	Bal_Acc	F1-Measure
STACKED RF^12^	0.739	0.544	0.935	0.739	0.622
SiGaAtCNN+ Input STACKED RF^11^	0.598	0.523	0.884	0.704	0.557
MDNNMD (Sp: 99%)^8^	**0.875**	0.200	**0.990**	0.595	0.326
MDNNMD (Sp: 95%)^8^	0.749	0.450	0.950	0.700	0.562
ChoqFuzGCN (proposed)	0.630	**0.666**	0.871	**0.769**	**0.647**

Significant values are in bold.

### Validation

The viability of the proposed model has been validated using the The Cancer Genome Atlas Program for Breast Cancer (TCGA-BRCA) dataset, which has been downloaded from https://xenabrowser.net/datapages/. A total of 1035 breast cancer patients’ private information has been utilized for the investigation. This dataset encompasses information on copy number variants, cancer-related gene expression, and clinical details. The data is preprocessed following a similar methodology as discussed in the “Dataset” section. Similarly, as described in the “Data preprocessing” section, the data is preprocessed. The TCGA-BRCA dataset consisted of 239 samples categorized as short-time survivors and 796 samples as long-time survivors. With the preprocessed and labeled version of the TCGA-BRCA dataset, the ChoqFuzGCN model is trained and validated using tenfold cross-validation. The observed findings of the model, along with those of other approaches, have been presented in Table 5. From the table, it is evident that ChoqFuzGCN has exhibited superior performance in terms of Mathews correlation coefficient, Precision, Balanced Accuracy, and F1-Measure compared to other techniques. In the case of dealing with an imbalanced dataset, the utilization of ChoqFuzGCN has led to an improvement in Balanced Accuracy. This indicates that our suggested method serves as a better strategy for handling imbalanced data.

**Table 5:** Performance metrics of ChoqFuzGCN, SVM (RBF kernel), RF, LR models on TCGA-BRCA dataset.

Metrices	Acc	Mcc	Pre	Sn	Sp	Bal_Acc	F1-Measure
GCN-RF	**0.738**	0.126	0.394	0.184	**0.905**	0.545	0.246
GCN-RBF	0.607	0.159	0.311	**0.555**	0.619	0.586	0.399
GCN-LR	0.613	0.152	0.308	0.544	0.633	0.589	0.393
GCN-Majority Voting	0.640	0.190	0.320	0.540	0.651	0.596	0.402
ChoqFuzGCN	0.715	**0.230**	**0.396**	0.440	0.798	**0.619**	**0.415**

Significant values are in bold.

### Statistical significance test

The validity of this prediction task can be considered established when the findings from tests such as the t-test and ANOVA are statistically significant. To validate the proposed study, the STACKED RF model and ChoqFuzGCN model were executed 10 times on the METABRIC dataset. Subsequently, t-test^35^ was performed on the recorded Balanced Accuracy, Sensitivity, Specificity, and F1-Measure using the *scipy* library. The results have shown that the obtained t-values (t-test) of 9.45, 26.4, 38.5, and 3.7 are statistically significant in terms of Balanced Accuracy, Sensitivity, Specificity, and F1-Measure, respectively, and p-values of 0.00 were obtained for all the mentioned metrics. The t-values of the ANOVA test are 438.54, 501.23, 612.23, and 396.12 for Balanced Accuracy, Sensitivity, Specificity, and F1-Measure, respectively, and the p-values are 0.00. These t-values and p-values indicate that our suggested model holds statistical significance and will be beneficial for the estimation of breast cancer survival.

## Discussion and conclusion

In the proposed work, we have introduced a Graph Convolutional Network-based Choquet Fuzzy Ensemble model to correctly predict breast cancer prognosis. The obtained Accuracy, Matthews correlation coefficient, Precision, Sensitivity, Specificity, Balanced Accuracy, and F1-Measure values from this model are 0.820, 0.528, 0.630, 0.666, 0.871, 0.769, and 0.647, respectively. If uni-modal CLN modality is considered, it is capable enough to provide a high Sp value. In other words, we can say that the CLN modality contains certain clinical features about the patients which help the model to correctly identify the long-term survivors belonging to class 0. But, this high Sp value (0.863) is obtained at the cost of low Sensitivity (Sn) value (0.316) means miss-classification of short-term survivors. The biological interpretation of this case suggests that the oncologist can provide a less aggressive treatment plan for the long-term survivors while relying on the CLN modality only. Unfortunately, they might end up following a similar treatment plan for short-term survivors also due to the miss-classification of short-term patients as long-term patients. Now considering the bi and multi-modal combinations where CLN modality is present achieves better Sn values while maintaining comparable or much higher Sp values, respectively when compared with CLN (uni-modal) modality. When using multi-modal approaches, the phenomenon of increased Sensitivity without losing Specificity occurs where the supplementary modalities provide complementary information for positive instance detection. Hence, incorporating additional modalities is necessary for a better survival prognosis for both classes. It is evident that the integration of all three modalities in the proposed model has led to improved performance and achieved better results across various metrics. The expanded investigation combining different modalities has been done to enhance the overall study (see Table 2). From that, it can be concluded that the proposed approach containing gene expression, clinical, and copy number alteration data is better than all other combinations of modalities for breast cancer prognosis prediction. Further, it can be seen that the proposed novel model is performing better than other state-of-the-art methods as well as other classifiers (depicted in Table 3 and 4). This finding highlights the previously unexplored improvement in predictive performance achieved by leveraging the sharing of underlying structural information between samples through Graph Convolution. Additionally, the Choquet Fuzzy ensemble enhances prediction performance by capitalizing on the inherent uncertainty present in the decision scores. Here, it is observable that multi-modal approaches continue to better performance. In future work, Additional omics data, such as DNA methylation, miRNA expression, and pathology image data, will be integrated to broaden the scope of the inquiry. Furthermore, this model has been examined for breast cancer prognostic prediction. However, there is potential for using this approach to predict additional diseases. This will be introduced in the future. The study also has certain limitations that need to be addressed in future work. Firstly, the feature extraction step may have overlooked important genes, and to overcome this, alternative techniques like Recursive Feature Elimination (RFE) will be incorporated. Additionally, improving Sensitivity, a crucial metric in this medical problem remains a challenge. To address this, adjustments to the decision threshold will be made to prioritize Sensitivity over Specificity. In this work, it is assumed that the training data is pure and devoid of noise. Thus, this model could suffer from adversarial attacks with noisy data. A federated framework-based study could be incorporated in the future to address this issue. Further, the study aims to improve the model’s overall performance and reliability in the future.

## Data Availability

The dataset and the source code of this proposed work are available at https://github.com/SusmitaPalmal/ChoqFuzGCN.
